# The Role of Id2 Protein in Neuroblatoma in Children

**DOI:** 10.1007/s12253-015-9908-9

**Published:** 2015-03-14

**Authors:** Aleksandra Wieczorek, Walentyna Balwierz

**Affiliations:** Department of Pediatric Oncology and Hematology, Polish-American Institute of Pediatrics, Jagiellonian University Medical College, Krakow, Poland

**Keywords:** Neuroblastoma, Id2 protein, Prognostic factor

## Abstract

Id (DNA binding and/or differentiation) proteins occur physiologically during ontogenesis and negatively regulate the activity of other helix-loop-helix (HLH) proteins. Id2 protein causes block of cells differentiation in the S phase of the cell cycle and regulates the activity of Rb protein. The role of Id2 protein in physiological cell cycle progression and in neuroblastoma (NBL) pathogenesis was proposed by Lasorella. The aim of the study was evaluation of Id2 expression and its prognostic significance in NBL cells coming from primary tumors and evaluation of its prognostic significance, and correlation of Id2 expression with known prognostic factors. Sixty patients with primary NBL treated from 1991 to 2005 were included in the analysis. We found 50 patients with high and 10 patients with low intensity of Id2 expression. The median percentage of NBL cells with Id2 expression was 88 %. We found no correlation between the number of NBL cells or the intensity of Id2 expression and OS and DFS. In patients with stage 4 NBL, almost all patients had high expression of Id2 and it was significantly more common than in other disease stages (*p* = 0,03). We found no correlation between Id2 expression and other known prognostic factor in NBL patients. We assume that Id2 is not prognostic factor. However, due to its abundant expression in most of NBL cells and its role in cell cycle, it may be potential therapeutic target. Exact knowledge of expression time may be helpful in explaining mechanisms of oncogenesis.

## Introduction

Id (DNA binding and/or differentiation binding) proteins have a helix-loop-helix (HLH) structure (Table 1). They occur physiologically during ontogenesis and negatively regulate the activity of other HLH proteins. Unlike other proteins from the HLH family, Id proteins (Id1 – Id4) lack the DNA binding basic domain. It results in the block of the formation of functional dimers of transcription regulators with tissue-specific differentiation regulators, so the cells differentiation is blocked and the cells come into the phase S of the cell cycle [1–6]. As the only protein from the HLH family, Id2 can also physically interact with Rb protein and prevent its antiproliferative activity. As an effect, Id2 can simultaneously control cell differentiation and cell cycle progression [1–3, 5, 7, 8]. It is assumed that, in the condition of abundant Id2 expression, the increased amount of unphosphorylated pRb protein is not sufficient for inhibition of cell cycle [2, 3, 7, 8]. During early stages of cell differentiation all proteins from Id family are expressed in many tissues and organs. The time and the level of expression depend on tissues/organs and the stage of differentiation [5, 9–11]. In the postnatal period Id2 expression is described in some postmitotic neurons of the central nervous system [9, 12, 14]. Id2 expression, as well as MYCN expression, is not physiologically found in normal adrenal glands during postnatal period [15]. Id proteins are necessary for maintaining the immaturity of progenitor cells and their proliferation as long as the established point in their development is reached [14, 16, 17]. This is especially important in control of nervous system cells differentiation and coordination of differentiation with definitive termination of cell proliferation and irreversible block of cell cycle progression [3, 8, 9, 12, 14, 18–20]. There is also a hypothesis concerning the influence of Id2 proteins on primary neoplastic cells (tumor stem cells). In such a case the presence of Id2 protein even in the single neoplastic cell could confirm its crucial role in tumor development [21, 22]. The high level of Id2 protein was found in some NBL cell lines with MYCN amplification. Features characteristic for cells with high activity of Myc family protein, as ability to enter the cell cycle without the presence of growth factors and ability of cooperation with ras protein are supposed to be dependent on Id2 protein function [14, 23]. Lasorella et al. found that excessive Id2 expression in neuroblastoma (NBL) cells is responsible for neoplastic transformation of precursor cells and its constant expression is required for keeping malignant character of NBL cells [8, 15]. As Id2 expression is especially crucial for normal nervous cells development the neoplastic cells coming from nervous system might be more sensitive for abnormal Id2 expression [12, 13, 15, 18].

## Material and Methods

The aim of the study was evaluation of Id2 expression in neuroblastoma cells coming from primary tumors stored as paraffin blocks taken from patients at diagnosis, evaluation of its prognostic significance and correlation between Id2 expression and known prognostic factors. Sixty patients were included in the analysis, treated for NBL in the Department of Oncology and Hematology in Krakow from 1991–2005. Observation was finished in June 2012. The characteristics of patients are presented in Table 2. Analysis of the significance of Id2 protein expression was performed both for the whole group of patients and for subgroups defined according to chosen established prognostic factors, as age, clinical stage of disease and MYCN amplification (Table 3).

**Table 1:** Abbreviations list

DFS	disease free survival
HLH	helix-loop-helix
Id	DNA binding and/or differentiation protein
NBL	neuroblastoma
OS	overall survival
RT-PCR	reverse transcription polymerase chain reaction

**Table 2:** Patient characteristic

Parameters		Number of patients (%)
Sex	Boys	34 (57.0)
	Girls	26 (43.0)
Age (months)	Range	0,3 – 169
	Median	24,5
	Number of patients >12 months of age	41 (68.3)
	Number of patients <12 months of age	19 (31.7)
Stage	1	0 (0.0)
	2	6 (10.0)
	3	17 (28.3)
	4	31 (51.7)
	4 s	6 (10.0)
MYCN amplification	Present	12 (20.0)
	Absent	46 (76.7)
	No result	2 (3.3)

**Table 3:** Treatment results depending on intensity of Id2 staining and number of Id2 positive cells in the whole group of patients and defined subgroups

	Intensity of Id2 staining	Number of Id2 positive cells
	*p* value	
Age at diagnosis	0.39	0.53
Presence of MYCN amplification	0.33	0.73
Whole group of patients		
Deaths	0.19	
OS	0.3	
Therapy failures	1.0	
DFS	0.15	
Patients over 1 year of age		
Deaths	0.35	0.79
OS	0.55	
Therapy failures	0.58	0.07
DFS	0.53	
Patients under 1 year of age		
Deaths	0.57	0.06
OS	0.42	
Therapy failures	0.21	0.86
DFS	0.19	
Amplification of MYCN		
Deaths		0.75
Therapy failures		0.51
No amplification of MYCN		
Deaths	0.17	0.38
OS	0.2	
Therapy failures	0.15	0.02*
DFS MYCN	0.13	

*higher number of cells in children without therapy failures

Id2 expression was evaluated with immunohistochemistry. Staining was performed with rabbit anti-Id2 antibodies (Zymed Laboratories Inc.) on silanized slides. According to manufacturer guidelines, the optimal dilution of primary antibody was experimentally established as 1:100 and the time of incubation as 45 min. The detection system was based on peroxidase (EnVision + System-HRP®, DacoCytomation) for use with rabbit primary antibodies, for evaluation in light microscopy of paraffin embedded tissue. As the positive control NBL cell line SKNSH was used with known high Id2 expression. As the second control human testis was used, embedded in paraffin. The double control system was chosen because we wanted to compare staining procedures in the samples prepared under the same conditions in our laboratory (testis tissue, unlike the cell line, underwent the same deparaffinization procedures and antigen unblocking). In negative control, no primary antibody was added. The percentage of cells with Id2 expression was evaluated in the light microscopy. The intensity of Id2 staining was evaluated by comparison with staining intensity of the positive control.

Estimations of the survival were performed using a Kaplan-Meier method and compared with a log-rank test. The comparisons between groups were performed by a chi-square test (with Fisher’s exact test if necessary), Student’s *t* test and Pearson correlation test. For all analyses the p values less than 0.05 (*p* < 0.05) were considered as statistically significant. Statistica® software packages were used for statistical analysis.

The influence of intensity of Id2 expression and the percentage of NBL cells with Id2 expression on mortality and therapy failures (initial progression or relapse) was evaluated.

## Results

**Fig. 1:**
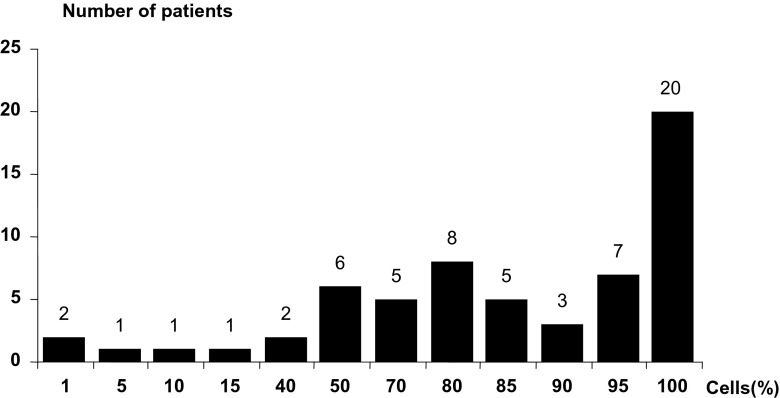
Percentage of cells with detectable Id2 protein expression in the whole group of patients

Among 60 NBL patients evaluated, we found 50 patients with high and 10 children with low intensity of Id2 expression. The percentage of NBL cells with the Id 2 expression was 1–100 % (median 88 %, mean 77.5 %). In 53 patients (88 %) the Id2 expression was found in more then 50 % of cells and in 28 (46.7 %) – in over 90 % (Fig. 1).

**Fig. 2:**
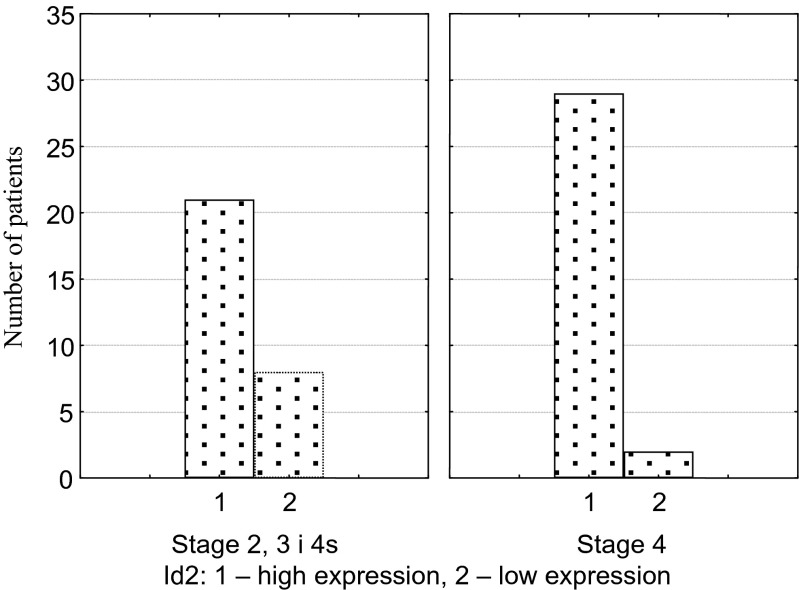
Number of patients with high and low Id2 protein expression in children with stage 4 and other stages (2, 3 and 4 s) of neuroblastoma

As the main aim of the study was the evaluation of Id2 as a prognostic factor, we decided to exclude from the overall survival analysis the patients whose death was not caused by NBL. Deaths occurred in 29 out of 50 patients (23 died of NBL) and 3 out of 10 patients (all caused by NBL) with high and low Id2 expression, respectively (chi-square, Fisher’s exact test, *p* = 0.19). Three-year OS was similar for both groups (0.68 and 0.7 in the groups with low and high Id2 expression, respectively). Five-year OS was higher in patients with low Id2 expression, but the results were not statistically significant (*p* = 0.3). Therapy failure defined as early progression or relapse occurred in 30 out of 50 children with high Id2 expression and 4 out of 10 children with low Id2 expression (chi-square, Fisher’s exact test, *p* = 1.0). No statistically significant differences were found (*p* = 0.15) for 3-year DFS (0.7 and 0.48) and 5-year DFS (0.7 and 0.4). In 41 evaluated patients over 1 year of age, the percentage of NBL cells with Id2 expression was 1-100 % (mean: 73.3 %, median: 85 %). In 17 patients (41.5 %) the percentage of Id2 positive cells was over 90 %. In children younger than 1 year of age (*n* = 19) the percentage of cells with Id2 expression was 50 %-100 % (mean 86 %, median 90 %). In 9 patients (47.4 %) Id2 expression was found in over 90 % of cells. In patients with stage 4 NBL, only 2 among 31 patients had low expression of Id2 – we did not perform comparative analysis. The percentage of Id2 positive cells was 1–100 % (mean 74 %, median 85 %). In patients with stage 4, in comparison to other stages, we found significantly more common high expression of Id2 (*p* = 0.03) (Fig. 2). The number of cells with high Id2 expression was not higher in patients in stage 4 (*p* = 0.46). The percentage of Id2 positive cells in patients in stage 2, 3 or 4 s was 1–100 % (mean 81.7 %, median 90 %). In this group of patients neither intensity of Id2 expression nor percentage of Id2 positive cells had influence on treatment outcomes (overall survival or disease relapse and progression). Among 12 patients with MYCN amplification, only 1 had low Id2 expression. The comparative analysis was not performed. The percentage of Id2 positive cells in patients with MYCN amplification was 40–100 % (mean 81.2 %, median 97.5 %). Percentage of Id2 positive cells had influence on neither NBL deaths (*p* = 0.75) nor therapy failure (*p* = 0.51) in this group of patients. In patients without MYCN amplification (*n* = 46) the percentage of Id2 positive cells was 1–100 % (mean 81.2 %, median 97.5 %). The intensity of Id2 expression was not different in patients with and without MYCN amplification (*p* = 0.33). In the group with no amplification, 15/37 children with high Id2 expression and 2/9 children with low Id2 expression died of NBL (*p* = 0.17). Therapy failures occurred in 22/37 and 3/9 children, respectively (*p* = 0.15). In patients with no MYCN amplification, 3-year and 5-year OS and DFS were higher in the group with low Id2 expression. However, the difference was not statistically significant (*p* = 0.2 for OS and *p* = 0.13 for DFS). Percentage of Id2 positive cells had no influence on survival (*p* = 0.39) and on therapy failures (*p* = 0.2).

Number of cells with Id2 expression and intensity of Id2 expression in cells had no statistically significant influence on mortality or therapy failures in the other groups of patients distinguished on the basis of age, stage or status of MYCN amplification. For the whole group and defined subgroups we did not find any correlation between the percentage of cells with Id2 expression and OS, DFS, levels of lactate dehydrogenase (LDH), ferritin or neurospecific enolase (NSE).

## Discussion

Extraordinary biological characteristics of NBL and diverse clinical outcome are the reasons why neuroblastoma is the focus of scientific interest [24–31]. One of the possible mechanisms responsible for oncogenesis is the dysfunction of proteins responsible for growth and differentiation of cells, as Id family proteins. They maintain stem cells pool of the nervous cells and their cytoplasmic sequestration promotes neural differentiation [32]. However, the results of studies concerning the possible meaning of Id2 in NBL remains unclear. In the study of Lasorella [15] they found correlation between expression of MYCN and Id2 protein with statistically significant influence of expression of Id2 in at least 25 % of cells on OS and DFS [3, 15]. Other studies did not confirmed these results. Vandesompele et al. [33] evaluated ID2, MYCN and MYC genes expression in NBL cell lines and did not confirm correlation between mRNA ID2 and either MYCN or MYC. There was no correlation between Id2 and patients’ prognosis. However, there was a trend for better prognosis in patients with Id2 level lower then the mean for the whole group. Alaminos et al. [34] evaluated Id2 in the group of 99 patients and 12 cell lines and did not reveal any correlation between MYCN and Id2 and it had no influence on prognosis. Both proteins and genes were evaluated by Wang et al. [35] in 170 patients and 10 cell lines and they were found in all evaluated tumors and cell lines. Id2 did not correlate with clinical patients’ characteristic and it was expressed independently of MYCN status; these results are consistent with the ones obtained in our study. However, we found that almost all patients with MYCN amplification had high Id2 expression, as had the patients in stage 4 NBL. The differences concerning correlation of Id2 with MYCN can be the result of different attitude to MYCN interpretation (amplification vs prognosis) and methods of Id2 evaluation (protein vs gene expression) [36, 37]. Differences in cell culture conditions can be also partially responsible for the observed inconsistent results [33]. It has been proved that Id2 expression in cell lines is induced by hypoxia, what can partially explain the described dedifferentiation of hypooxygenated NBL cells [38]. It should be also pointed that during normal embryogenesis the appropriate level of Id2 is crucial in the given time points, so probably for correct interpretation of the significance of Id2 protein expression it would be necessary to know the exact time point of its expression [39].

In our study we found expression of Id2 protein in all evaluated tumors, with 83.3 % of patients with high expression in most of the cells. As neither the exact level nor the meaning of time of Id2 expression is confirmed to be informative, we assumed that each observed expression of Id2 will be defined as positive. There are some studies indicating the existence of neoplastic stem cells, in which the mechanisms connected with Id2 expression can play a potential role in creating and maintaining malignant phenotype of the cell. In our material the intensity of Id2 expression was comparable in all analyzed groups; however, and lack of statistical significance may be the result of a small number of patients. After analysis of the whole group and subgroups it cannot be confirmed that Id2 expression has prognostic significance in NBL. In most of univariate analyses, neither level of expression nor number of cells with Id2 expression had statistically significant influence on OS and DFS. However, almost all children with MYCN amplification had high intensity of Id2 expression and more children with strong Id2 expression were in stage 4 of disease.

It has been proved that inhibition of Id2 function is possible in vitro, and it results in reduction of tumorigenic properties in neuroblastoma cells and promotion of cells differentiation [40–42]. Additionally, it has been proved that retinoic acid treatment is associated with negative regulation of ID2 mRNA [43]. Because of common expression of Id2 in neuroblastic tumors and its known role in nervous system development, it may be a potential therapeutic target and it is necessary to continue work explaining meaning of the level and time of its expression in NBL cells.
